# Supplementary material for: Identification and Functional Analysis of a Key Gene in the CHH Gene Family for Glucose Metabolism in the Pacific White Shrimp Litopenaeus vannamei
Source: Int J Mol Sci. 2025 May 12;26(10):4612. doi: 10.3390/ijms26104612 (PMC12111282; doi:10.3390/ijms26104612)
Supplement: Supplementary file 1 [file ijms-26-04612-s001.zip › Date S1-The amino acid sequence of the ORF region of the CHH gene of Litopenaeus vannamei.pdf]

Amino acid sequence of the ORF region of the CHH gene of *Litopenaeus vannamei*

>IOCAS.LVAN16181
MILQTTKPEADSLGIYLVCCRQHRADIRTPAGPHITKHAHHPKTSLHQAEFLAKRIHHHSHAAGNNNIPLTGRS
MTLLAAHHFKSPRQPCSSPASPRLAPDPKYRPPPPPARTLPPPEDALPLAPPAHSVTLPEDGIPSAPPAHCSSEAR
EHALSASRFRDQRLRTQGVSPKPLISPTCRTLTTAYIRRRPLGSVAEQTKSLEVAGSLLPIRVDAEEMIGVRLVRSAV
LVSLLLVFPASVLASWDGNEIPPSLPSSSESSPATSLAGAQTANKRSISFDSCTGVYDRELLVRLDRVCEDCYNLYRD
TDVAVECRSNCFHNEVFLYCVDYMYRPRQRNQYRAALQRLGK

>IOCAS.LVAN16180
MGFLQRRPLTAVPKPENMHSRHQTLTTAYIRRRPLGSVAEQTKSLEVAGSLLPIRVDAEEMIGVRLVRSAVLVSLL
LVFPASVLASWDGNEIPPSLPSSSESSPATSLAGAQTANKRSISFDSCTGVYDRELLVRLDRVCEDCYNLYRDTDVA
VECRSNCFHNEVFLYCVDYMYRPRQRNQYRAALQRLGK

>IOCAS.LVAN16186
MKDADDRLYKATSTWLRRRADEEPRSRRFSPPDSSRRRGNDWGSTVFPASVLASWDGNEI
PPSLPSSSESSPATSLAGAQTANKRSISFDSCTGVYDRELLVRLDRVCEDCYNLYRDTDVAVECRSNCFHNEVFLYC
VDYMYRPRQRNQYRAALQRLGK

>IOCAS.LVAN16184
MQDADDRLYKATSTWLRRRADEEPRSRRFSPPDSSRREEMIGVRLCFPASVLASWDGNEILRPCLPPQNPLLRP
PSGAQTANKRSISFDSCTGVYDRELLVRLDRVCEDCYNLYRDTDVAVECRSNCFHNEVFLYCVDYMYRPRQRNQ
YRAALQRLGK

>IOCAS.LVAN16185
MILQTTKPEADTVKANLYNASASTPYPAKHTVTQSQLLTPQHLSRLLPPHRAISGQNITKDADHPKTSLHRAEFL
AKRIHHRSHAAGSNNTPLTGRAMTLLATHCFKSTRQPAAPSFSSLAPHQNPTLTTAYIRRRPLGSVAEQTKSLEVA
GSLLPIRVDAEEMIGVRLVRSAVLVSLLLVFPASVLASWDGNEIPPSLPSSSESSPATSLAGAQTANKRSISFDSCTG
VYDRELLVRLDRVCEDCYNLYRDTDVAVECRSNCFHNEVFLYCVDYMYRPRQRNQYRPPCRGSASRRFLSGHTF
YGDARAMISRRPSQRRTVLGRRGGGDRAISPSVLPGNVLKI

>IOCAS.LVAN16183
MYLSAPPPARTLPPPEDALPSAPPAHSVTPPEDGILQRRPLTAVPKPENMHSRHQTLTTAYIRRRPLGSVAEQTKS
LEVAGSLLPIRVDAEEMIGVRLVRSAVLVSLLLVFPASVLASWDGNEIPPSLPSSSESSPATSLAGAQTANKRSISFD
SCTGVYDRELLVRLDRVCEDCYNLYRDTDVAVECRSNCFHNEVFLYCVDYMYRPRQRNQYRAALQRLGK

>IOCAS.LVAN16174
MKDADDRLYKATSTWLRRRADEEPRSRRFSPPDSSRRRGNDWGSTAVLVSLLLVFPASVLASWDGNEIPPSLPS
SSESSPATSLAGAQTANKRSISFDSCTGVYDRELLVRLDRVCEDCYNLYRDTDVAVECRSNCFHNEVFLYCVDYMY
RPRQRNQYRAALQRLGK

>IOCAS.LVAN16178
MQDADDRLYKATSTWLRRRADEEPRSRRFSPPDSSRRRGNDWGSTAVLVSLLLVFPASVLASWDGNEIPPSLPS
SSESSPATSLAGAQTANKRSISFDSCTGVYDRELLVRLDRVCEDCYNLYRNTDVAVECRSNCFHNEVFLYCVDYMY
RPRQRNQYRAALQRLGK

>IOCAS.LVAN16179
MFASRMVWSALVLSLMVALAASAATWDRSFRDEEEPPKFLPPSSPDSSAVALPRTLPLPADEDHSLSKRSGYYN
SCTGVYDRELIARLDRVCEDCYNLYRDVEVAVGCRKGCYHNEVFLYCVDYMFRPRQRNQYRAALQKLGK

>IOCAS.LVAN16176
MVAVQLAVLMCMLLAVPAAITTHDNTNELPKFLLSSPGDSLTSGQSLIKRTTSFSSCTGVYDRELLARLDRVCED
CYNLYRDVGVAAECRSNCFHNEVFLYCVDYMYRPRQRNQYRAALQRLGK

>IOCAS.LVAN16175

MAAVGPMRAAVLVSLLVAIPASATTSGDENEIPTLLRHTQKVSPVSSFAGAHSLHKRSLSFRSCTGVYDRELLARL
DRVCEDCYNIYRDVGVAAECRSDCFHNEVFLYCVDYMYRPRQRNQYRAALQRLGK
>IOCAS.LVAN16177
MILQTTKPEADSLGRRPLGSVAEQTKSLEVAGSLLPIRVDAEEMIGVRLVRSAVLVSLLLVFPASVLASWDGNEIPP
SLPSSSESSPATSLAGAQTANKRSISFDSCTGVYDRELLVRLDRVCEDCYNLYRDTDVASNAGATVSTTRYSCTAST
TCTGLAKGTSTGPPCRGSASRRFLSGHTFYGDARAMILQTTKPEADSLGPSSFYSPLFSLSIPSPHPPAVPLSPPPH
YSLFLLPLTPIRSFPLLSLSVPSKLRLHFLPDHMQCPSRFSNPLCSLSPYPPFSPPLPHPSTPALPHPLPPLPPSLPPPL
HLLLPPSSLPCPPSSPPLSSLIHLPIPSLLPPALPPSLLPPPPASLLPPALPPPPPLALLLPLSSLLPPKRTRPSAAAISPP
>IOCAS.LVAN16182
MKDADDRLYKATSTWLRRRADEEPRSRRFSPPDSSRRRGNDWGSTGAFSCPGIPAASVPASVLASWDGNEIPP
SLPSSSESSPATSLAGAQTANKRSISFDSCTGVYDRELLVRLDRVCEDCYNLYRDTDVASNAEQTKSLEVAGSLLPI
RVDAEEMIGVRLVRSAVLVSLLLVFPASVLASWDGNEIPPSLPSSSESSPATSSREPRPQTSAAYPSTRARASTTAN
SLSNCFHNEVFLYCVDYMYRPRQRNQYRAALQRLGK
>IOCAS.LVAN17415
MWSAAIVTLLVAAAACASSWERSLEMEGQTSEFLPSFPQSPSLLSSAADHSLRKRSIFDHSCTGVFDRELIGRLN
RVCDDCYNVFRDTDVATGCRSNCFYNRMFLQCLVYLFPPRFRNQYKAAVQMVGKARRWFVEPDAGSRIFFDD
FRRQMDQPHRLYIKGTAAFWSQSNCLRGRSHSRQLLIL
>IOCAS.LVAN07719
MTAFRMVWSMLLASLLLLLAASSAAPADALSAPAAGLTKRSLFDPSCTGVFDRQLLRRLRRVCDDCFNVFREPN
VSTECRSNCYNNEVFRQCMEYLLPPHLHEEHRLAVQMVGK
>IOCAS.LVAN07718
MMGWRSAVSAQTDTQNHHISSAKRCSIFCANYACLPYYVVSDNGLFAAAARGVVRCPRRRLIRPYHGLTKRSL
FDPSCTGVFDRQLLRRLRRVCDDCFNVFREPNVAIDCRENCYNNEVFRQCMAYVVPANLHDEHRQAVQMVG
K
>IOCAS.LVAN17414
MIALRLMAVTLLVALAASTTWARSFNKRANFDPSCTGVYDRELLGRLSRLCDDCYNVFREPKVATECRSNCFYN
PVFVQCLEYLIPADLHEEYQALVQTVGK
>IOCAS.LVAN19516
MCFSSKMAVALVVVVACSTTWARSAEESSSPVASLIRGRSLSKRANFDPSCTGVYDRELLGRLSRLCDDCYNVFR
EPKVATECRSNCFYNPVFVQCLEYLIPADLHEEYQALVQTVGK
>IOCAS.LVAN17417
MVSFLSLRMVCSAALVSLLVLALSSRSAFARSVDGVGRLEKLLSSSSSSSGSSSPLDALGGDHSVNKRDTFDHSCK
GIYDRELFRKLDRVCEDCYNLYRKPYVATECKSNCFVNKRFNVCVADLRHDVSRFLKMAKFLRYP
>IOCAS.LVAN18220
MARKHPCHGDKISPQHPPTAFAFRRAVAEADFAGSLQRQLERKMVVPKALHFDKIFQKRLMLFLVLMICQQGY
ASFIKVRPNTLREFQFLKCQGEFNKAQYVSLSHVCEDCHNLYRQPEILTECKANCFQNTLFPTCVSLLMLDRHED
DLNKKVALISGQEL
>IOCAS.LVAN22614
MDNKYFCAAVVSHKLRSSLPQIAFVSASVLLLVAVLASHNGVHARSVVPEGLQELEIPRQESDMFAVRRKRQVF
DASCKGVYDRGLWAKLNNACLDCQNIYRGNPAIEGECR
>IOCAS.LVAN19748
MQWANVRKANQEKKDLSQSPIWPLIGSSIRPVKVSIHQVNCSATVMRAAFPYKGKPGRWKASAISHHHVRSS
GPLSAVMSGCAMWVILALTASCSVMCHARIMDPGHSRPPFHPPSPPSPSTSSSSASALRVAKRDVFDPSCKGIY
NRAIWAKLNRACEDCQNLFRDEMGIYESCREKCFDTKIFPACVIELSLNLNEYMFEAELIREL

>IOCAS.LVAN19742
MQWANVRKANQEKKDLSQSPIWPLIGSSIRPVKVSIHQVNCSATVMRAAFPYKGKPGRWKASAISHHHVRSS
GPFSAVMSGCAMWVILALTASCSVMCHARIMDPGHSRPPFHPLSPPSPSTSSSSASALRVAKRDVFDPSCKGIY
NRAIWAKLNRACEDCQNLFRDEMGIYESCREKCFDTKIFPACVIELSLNLNEYMFEAELIREL
>IOCAS.LVAN19744
MRKSPAGGSEAIRRFLTEYNESAKHLLQYKVECGQYPVPGYCGQDKVGRTAHGFRDGQERIAFPLDTLFNLNAI
VASALHLAMINTVPILQTAGERKDLELRCRGRCDIIFSYQRTLGTYEHRMFLETAHAYQDQYMFAITTYTAGTLGL
SPTAKDQEVGHSLWVVHCADKTPEEECVVSSYRRKMVLSQLVQFLAALQLPKWHEIRLSPGSREVATPYDHTE
MPWILLLHDSASRSRVMTLVPHLAQLLHGTAATLTFDLEQEGVEVLADLGLSASNIVAPALAILQPPHLRKPTATV
PLLNDYDDPLEWLNDQLDDLQIQPVLSKEEQERESLTKRDTHSIPFPFPDTHILLPYYSLSLTYIPFSPIPFSYPEVRT
SLPYLSLTLRPATDHRLFHSRVATGRRTGARNGDPVSNILLQHLDSIVPQLHELGSHASLYRVNCFDWPKVCDAN
GISTYPVLRLHPKGANNITYDGAISGAGILKAILLCEGSTPLEVTSGEVLEELLRLSPSLHPALALPSPPAVAVGVFAT
LRDAAGVTQAAAILRGTHLVARHISPAATSSMCGSRAGCVVVAKPHDRYQPRRILRDHLNDPSAIANVLRYATLP
VMGRLDPERYAALTQSGGAKADDLAPDQYHVILFLPAASSEFPSGGREPRDAALDAVGQVAAEVAGPDLTFSW
LSMYGWTAGHLAVLRPRRAQPRPGRREPPRQDGHRVPSRRPGPFVSPAVAAGGQVTLADNAWEPSLAGIDY
LRLLEEDATTSSWTRCSPRGTVTGTSAARRATLSSILLPARPHPSSTLALTLSPLTLCLQEPSISVAKPRPIAFAETVYT
YDIGTGVLQRPVGRSYALPAREPRMQWANVRKANQEKKDLSQSPIWPLIGSSIRPVKVSIHQVNCSATVMRAA
FPYKGKPGRWKASAISHHHVRSSGPLSAVMSGCAMWVILALTASCSVMCHARIMDPGHSRPPFHPPSPPSPS
TSSSSASALRVAKRDVFDPSCKGIYNRAIWAKLNRACEDCQNLFRDEMGIYESCREKCFDTKIFPACVIELSLNLN
EYMFEAELIREL
>IOCAS.LVAN05100
MGGTCPGRMGNREMYTKVDRVCEDCANIFRLPVLEGLCRDRCFYNEWFLLCLKAANREDEIENFRVWISILNA
>IOCAS.LVAN05101
MYRLAIRSWLPVMTVLFATSLFFDTASASPIDGTCPGRMGNREIYKKVDSVCKDCVNIFRLPELEGLCRDECFIN
DWFLFCAKAAKRMDEIENFRVWISILNA
>IOCAS.LVAN05098
MRTWLALVIVLVGTSLFVDTASASFTDGACRGIMGNREIYKKVERVCEDCTNIFRLPGLDGMCRDRCFYNEWF
LLCLKAANREDEIENFRVWISILNA
>IOCAS.LVAN05097
MQRILYKGGSRPTTALHSRRLRTPLSAPSSSAFVHLYAYTLMYRLAMRTWLVVILVVVGTSLFFDTASASLIHGTCR
GVMGNREIYEKVVRVCEDCTNIFRMPGLDGMCRDRCFYNEWFLLCLKAANREDEIENFRVWISILNAGQ
>IOCAS.LVAN05099
MYRPVIRIWLALVIAVVGASIFFDSASASFIDGSCRGVMGNREIYKKVVRVCEDCTNIFRLPGLDVMCRDRCFH
NEWFLLCLNAANREDEIENFKVWISILSAGQ
