# Supplementary material for: Identification and Functional Analysis of a Key Gene in the CHH Gene Family for Glucose Metabolism in the Pacific White Shrimp Litopenaeus vannamei
Source: Int J Mol Sci. 2025 May 12;26(10):4612. doi: 10.3390/ijms26104612 (PMC12111282; doi:10.3390/ijms26104612)
Supplement: Supplementary file 1 [file ijms-26-04612-s001.zip › Date S2-The amino acid sequences of 136 CHH proteins with CHH family domains and eight proteins related to glucose metabolism.pdf]

Demonstrated CHH associated with glucose metabolism in crustaceans (8)

>AAN86056.1 crustacean hyperglycemic hormone B1, partial [Penaeus vannamei]
EKLLSSSSSSSGSSSPLDALGGDHSVNKRDTFDHSCKGIYDRELFRKLDRVCEDCYNLYRK
PYVATECKSNCFVNKRFNVCVADLRHDVSRFLKMTKSLRYP
>AAN86057.1 crustacean hyperglycemic hormone B2, partial [Penaeus vannamei]
EKLLSSSSSSSGSSSPLDALGGDHSVNKRDTFDHSCKGIYDRELFRKLDRVCEDCYNLYRK
PYVATECKSNCYANFVFKQCLDDLLMVDAIDEYVNTVQLVGK
>AWX63582.1 hyperglycemic hormone-like peptide precursor [Penaeus monodon]
MVSFLSLRMVCSAALVSLLVLALSSRSAFARSVDGVGRLEKLLSSSSSSSSGSSSPLLALGG
DHSVNKRDTFDHSCKGIYDRELFRKLDRVCEDCYNLYRKPYVATECKVNCFVNQRFSNCV
ADLRYDVNRFEKMAMFLRYS
>BEG56311.1 crustacean hyperglycaemic hormone isoform 1 [Panulirus japonicus]
MWLAKVVVVAVVLMSSGVTGRSSSGLVRLEKLLSSKSSSSTPLSLLSADHNVNKRAVFDQ
SCKGVYDRSLFQKLDLVCDDCYNLYRKPYVATGCRENCYGNLVFRQCLDDLLLLDVVDE
YVASVQSVGK
>BEG56312.1 crustacean hyperglycaemic hormone isoform 2 [Panulirus japonicus]
MLAYRTLCVLVVVVVGVVRARSADGMARMEKLFSADTSSTATALDAAAEHSVNKRAVF
DQSCKGVYDRSLFGKLDRVCDDCYNLYRKHYVSTGCRKNCYGNLVFRQCLDDLLLLDV
VDEYVASVQSVGK
>AFM35652.1 crustacean hyperglycemic hormone [Scylla paramamosain]
MSTFTSVIQMAVLVACIAMATLPHTQGRSADGFGRMGRLLASLKADSLGPVQDYGVEGA
AHPLEKRQTFDSSCKGVYDRAIFSELEHVCNDCYNLYRTSRVASGCRSNCYSNVVIRQCM
EDLLLMDNFEEIARKIQMVGKK
>AJD81303.1 crustacean hyperglycemic hormone [Neohelice granulata]
MVTYRMTSTVALVVVVALGASILILPHAHARSAEGFGRMERLLSQIRGGSDSSAALGEMR
VAGEGPAGHPLEKRQIYDRSCKGIYDRSLFSKLEHVCDDCYNLYHTSHVASGCRENCYSN
LVFRQCMDDLLLMDMFDEYAKAIQVIGRKKK
>AAQ75760.1 hyperglycemic hormone precursor [Scylla olivacea]
MSALTSIMQMAVLVACITMATLPDTQARSAEGFGRMGRLLASLKADSLGPVQDFGVEGA
AHPLEKRQIFDSSCKGVYDRAIFNELEHVCNDCYNLYRTSHVASGCRSNCYSNVVIRQCM
EDLLLMDNFEEIARKIQMVGKK

Amino acid sequence of 84 CHH proteins were retrieved from NCBI database (2023.08)

>AAC60516.1 MIH-like, partial [Penaeus vannamei]
LEKLLSSSSSSSGSSSPLDALGGDHSVNKRDTFDHSCKGIYDRELFRKLDRVCEDCYNVFR
EPKVATECKSNCFVNKRFNVCVADLRHDVSRFLKMANSALS
>AAK69346.1 MIH, partial [Penaeus vannamei]
LSSSSSSSGSSSPLDALGGDHSVNKRDTFDHSCKGIYDRELFRKLDRVCEDCYNLYRKPYV
ATECKSNCFVNKRFNVCVADLRHDVSRFLKMAKFLRYP
>AAN86054.1 CHH B, partial [Penaeus vannamei]
EKLLSSSSSSSGSSSPLDALGGDHSVNKRDTFDHSCKGIYDRELFRKLDRVCEDCYNLYRK
PYVATECKSNCFVNKRFNVCVADLRHDVSRFLKMAKFLRYP

>AAN86055.1 CHH B, partial [Penaeus vannamei]
EKLLSSSSSSSGSSSPLDALGGDHSVNKRDTFDHSCKGIYDRELFRKLDRVCEDCYNLYRK
PYVATECKSNCYANFVFKQCLDDLLMVDAIDEYVNTVQLVGK
>AAN86056.1 CHH B1, partial [Penaeus vannamei]
EKLLSSSSSSSGSSSPLDALGGDHSVNKRDTFDHSCKGIYDRELFRKLDRVCEDCYNLYRK
PYVATECKSNCFVNKRFNVCVADLRHDVSRFLKMTKSLRYP
>AAN86057.1 CHH B2, partial [Penaeus vannamei]
EKLLSSSSSSSGSSSPLDALGGDHSVNKRDTFDHSCKGIYDRELFRKLDRVCEDCYNLYRK
PYVATECKSNCYANFVFKQCLDDLLMVDAIDEYVNTVQLVGK
>AAR04348.1 Liv-MIH1 precursor [Penaeus vannamei]
MYRLAMKTWLAIVIVVVGTSLFFDTTSASFIDGTCRGVMGNRDIYKKVVRVCEDCTNIFR
LPGLDGMCRDRCFYNEWFLICLKAANREDEIEKFKVWISILNAGQ
>AAR04349.2 Liv-MIH2 precursor [Penaeus vannamei]
MYRLAIRSWLPVMTVLFATSLFFDTASASPIDGTCPGRMGNREIYKKVDSVCKDCVNIFRL
PELEGLCRDECFINDWFLFCAKAAKRMDEIENFRVWISILNA
>AAR11295.1 CHH A, partial [Penaeus vannamei]
TKRSLFDPSCTGVFDRQLLRRLRRVCDDCFNVFREPNVSTECRSNCYNNEVFRQCMEYLL
PPHLHEEHRLAVQMVGK
>ABD73291.1 MIH1 [Penaeus vannamei]
MYRLAMKTWLAIVIVVVGTSLFFDTTSASFIDGTCRGVMGNRDIYKKVVRVCEDCTNIFR
LPGLDGMCRDRCFYNEWFLICLKAANREDEIEKFKVWISILNAGQ
>ABD73292.1 MIH2 [Penaeus vannamei]
MYRLAIRSWLPVMTVLFATSLFFDTASASPIDGTCPGRMGNREIYKKVDSVCKDCVNIFRL
PELEGLCRDECFINDWFLFCAKAAKRMDEIENFRVWISILNA
>ABN11282.1 ITP [Penaeus vannamei]
MVSFLSLRMVCSAALVSLLVLALSSRSAFARSVDGVGRLEKLLSSSSSSSGSSSPLDALGG
DHSVNKRDTFDHSCKGIYDRELFRKLDRVCGDCYNLYRKPYVATECKSNCFVNKRFNVC
VADLRHDVSRFLKVAKFLRYP
>ACT76341.1 CHH0, partial [Penaeus vannamei]
TKRSLFDPSCTGVFDRQLLRRLRRVCDDCFNVFREPNVSTECRSVIHGFPFFLHFLHQYPL
HDISFIAYDTEGCLRHPSLTLITYFYRSNCYNNEVFRQCMEYLLPPHLHEEHRLAVQMVGK
>ADL27417.2 CHH0 [Penaeus vannamei]
MTAFRMVWSMLLASLLLLLAASSAAPADALSAPAAGLTKRSLSDPSCTGVFDRQLLRRLR
RVCDDCFNVFREPNVSTECRSNCYNNEVFRQCMEYLLPPHLHEEHRLAVQMVGK
>ADL27743.1 GIH, partial [Penaeus vannamei]
DCANIYRLPQLDGLCRNRCFNNQWFLMCLHSAKREAELGHFRLWISIL
>AFV95080.1 CHH0-like peptide precursor [Penaeus vannamei]
MVSFLSLRMVCSAALVSLLVLALSSRSAFARSVDGVGRLEKLLSSSSSSSGSSSPLDALGG
DHSVNKRDTFDHSCKGIYDRELFRKLDRVCEDCYNLYRKPYVATECKSNCFVNKRFNVCV
ADLRHDVSRFLKMAKFLRYP
>AFV95081.1 CHH0-like peptide 2 precursor [Penaeus vannamei]
MVSFLSLRMVCSAALVSLLVLALSSRSAFARSVDGVGRLEKLLSSSSSSSGSSSPLDALGG
DHSVNKRDTFDHSCKGIYDRELFRKLDRVCEDCYNLYRKPYVATECKSNCFVNKRFNVCV
ADFRHDVSRFLKMAKFLRYP

>AGX26044.1 VIH [Penaeus vannamei]
MRTWLLLAIVAVGASLANILDSNCRGAMGNRDMYRKVERVCEDCTNIYRLPQLDGLCRN
RCFNNQWFLMCLHSAKREAELGHFRLWISILNAGRPW
>AHJ11242.1 GIH [Penaeus vannamei]
MRTWLLLAIVAVGASLANILDSNCRGAMGNRDMYRKVERVCEDCTNIYRLPQLDGLCRN
RCFNNQWFLMCLHSAKREAELGHFRLWISILNAGRPW
>AJK31204.1 CHH [Penaeus vannamei]
MDNKIAFVSASVLLLVAVLASHNGVHARSVVPEGLQELEIPRQESDMFAVRRKRQVFDAS
CKGVYDRGLWAKLNNACLDCQNIYRGNPAIEGECRQNCFGTEVFYGCIKALKLPTKNYL
YFAEVLRES
>AJK31205.1 CHH [Penaeus vannamei]
MDNKIAFVSASVLLLVAVLASHNGVHARSVVPEGLQELEIPRQESDMFAVRRKRQVFDAS
CKGVYDRGLWAKLNNACLDCQNIYRGNPAIEGECRQNCFGTEVFYGCIKALKLPTKNYL
YFAEVLRES
>AMR70770.1 CHH [Penaeus vannamei]
MDNKIAFVSASVLLLVAVLASHNGVHARSVVPEGLQELEIPRQESDMFAVRRKRQVFDAS
CKGVYDRGLWAKLNNACLDCQNIYRGNPAIEGECRQNCFGTEVFYGCIKALKLPTKNYL
YFAEVLRES
>ATN45407.1 MIH-like protein [Penaeus vannamei]
MWAVPSSRQGSGHFSPKATRLMASPRRTPSILKKACQVALVAAVLYGLLTAPASARFIDDEC
VGAMGNRNIYEKVARVCDDCSNIFRLPNVGESCRRNCFYNEDFLWCIMASERHAEVEQF
NRWISILKAGRK
>BAM93361.1 CHH precursor [Penaeus vannamei]
MTAFRLMAVALVVVVACSTTWARSAEESSSPVASLIRGRSLSKRANFDPSCTGVYDRELLG
RLSRLCDDCYNVFREPKVATECRSNCFYNPVFVQCLEYLIPADLHEEYQALVQTVGK
>BBA57870.1 sinus gland peptide A precursor [Penaeus vannamei]
MLAYRTMWSAIMASLLLLLAASSAAPADALSAPAAGLGADHGLTKRSLFDPSCTGVFDR
QLLRRLRRVCDDCFNVFREPNVAIDCRENCYNNEVFRHCMAYVVPANLHDEHRQAVQM
VGK
>BBA57871.1 sinus gland peptide B precursor [Penaeus vannamei]
MIGVRLVRSAVLVSLLLVFPASVLASWDGNEIPPSLPSSSESSPATSLAGAQTANKRSISFDS
CTGVYDRELLVRLDRVCEDCYNLYRDTDVAVECRSNCFHNEVFLYCVDYMYRPRQRNQY
RAALQRLGK
>BBA57872.2 sinus gland peptide F precursor [Penaeus vannamei]
MVLQYMLSAALLVLAASSSPAAARSLDAAPSSASSGSHSLSKRSLFDPACTGIYDRQLLGK
LGRLCDDCYNVFREPKVATGCRSNCYYNLIFLDCLEYLIPSHLQEEHMSALQTVGK
>BBA57873.1 sinus gland peptide G precursor [Penaeus vannamei]
MTAFRLMAVALVVVVACSTTWARSAEGSSSPVASLIRGRSLSKRANFDPSCTGVYDRELLG
RLSRLCDDCYNVFREPKVATECRSNCFYNPVFVQCLEYLIPADLHEEYQALVQTVGK
>BBA57874.1 sinus gland peptide C precursor [Penaeus vannamei]
MTAFRMVWSMLLASLLLLLAASSAAPADALSAPAAGLTKRSLFDPSCTGVFDRQLLRRLR
RVCDDCFNVFREPNVSTECRSNCYNNEVFRQCMEYLLPPHLHEEHRLAVQMVGK
>BBC21005.1 sinus gland peptide A precursor [Penaeus vannamei]
MLAYRTMWSAIMASLLLLLAASSAAPADALSAPAAGLGADHGLSKRSLFDPSCSGVFDR

QLLRRLRRVCDDCFNVFREPNVAIDCRENCYNNEVFRQCMAYVVPANLHDEHRQAVQM
VGK
>BBC21006.1 sinus gland peptide A precursor [Penaeus vannamei]
MLAYRTMWSAIMASLLLLLAASSAAPADALSAPAAGLTKRSLFDPSCTGVFDRQLLRRLR
RVCDDCFNVFREPNVAIDCRENCYNNEVFRQCMAYVVPANLHDEHRQAVQMVGK
>BBC21007.1 sinus gland peptide B precursor [Penaeus vannamei]
MIGVRLVRSAVLVSLLLVFPASVLASWDGNEIPPSLPSSSESSPATSLAGAQTANKRSISFDS
CTGVYDRELLVRLDRVCEDCYNLYRDTDVAVECRSNCFHNEVFLYCVDYMYRPRQRNQY
RAALQRLGK
>BBC21008.1 sinus gland peptide F precursor [Penaeus vannamei]
MVLQYMLSAALLVLAASSSPAAARSLDAAPSSASSGSHSLSKRSLFDPACTGIYDRQLLGK
LGRLCDDCYNVFREPKVATGCRSNCYYNLIFLDCLEYLIPSHLQEEHMSALQTVGK
>CAA68067.1 CHH0, partial [Penaeus vannamei]
AGLTKRSLFDPSCTGVFDRQLLRRLRRVCDDCFNVFREPNVSTECRSNCYNNEVFRQCME
YLLPPHLHEEHRLAVQMVGK
>QBS36529.1 MIH [Penaeus vannamei]
MWAVPSSRQGSGHFSPKATRLMASPRRTPSILKKACQVALVAAVLYGLLTAPASARFIDDEC
VGAMGNRNIYEKVARVCDDCSNIFRLPNVGESCRRNCFYNEDFLWCIMASERHAEVEQF
NRWISILKAGRK
>QEE04599.1 CHH isoform 1 [Penaeus vannamei]
MFASRMVWSALVLSLMVALAASAATWDRSFRDEEEPPKFLPPSSPDSSAVALPRTLPLPAD
EDHSLSKRSGYYNSCTGVYDRELIARLDRVCEDCYNLYRDVEVAVGCRKGCYHNEVFLY
CVDYMFRPRQRNQYRAALQKLGK
>QEE04600.1 CHH isoform 2 [Penaeus vannamei]
MVAVQLAVLMCMLLAVPAAITTHDNTNELPKFLLSSPGDSLTSGQSLIKRTTSFSSCTGVYD
RELLARLDRVCEDCYNLYRDVGVAAECRSNCFHNEVFLYCVDYMYRPRQRNQYRAALQ
RLGK
>ROT60578.1 hypothetical protein C7M84_011781, partial [Penaeus vannamei]
VRSAVLVSLLLVFPASVLASWDGNEIPPSLPSSSESSPATSLAGAQTANKRSISFDSCTGVYD
RELLVRLDRVCEDCYNLYRDTDVAVECRSNCFHNEVFLYCVDYMYRPRQRNQYRAALQR
LGK
>ROT60579.1 hypothetical protein C7M84_011782 [Penaeus vannamei]
MIGVRLVRSAVLVSLLLVFPASVLASWDGNEIPPSLPSSSESSPATSLAGAQTANKRSISFDS
CTGVYDRELLVRLDRVCEDCYNLYRDTDVAVECRSNCFHNEVFLYCVDYMYRPRQRNQY
RAALQRLGK
>ROT60580.1 CHH 1 precursor [Penaeus vannamei]
MKDADDRLYKATSTWLRRRADEEPRSRRFSPPDSSRRRGNDWGSTAVLVSLLLVFPASVL
ASWDGNEIPPSLPSSSESSPATSLAGAQTANKRSISFDSCTGVYDRELLVRLDRVCEDCYNL
YRDTDVAVECRSNCFHNEVFLYCVDYMYRPRQRNQYRAALQRLGK
>ROT60581.1 CHH 1 precursor [Penaeus vannamei]
MAAVGPMRAAVLVSLLVAIPASATTSGDENEIPTLLRHTQKVSPVSSFAGAHSLHKRSLSFR
SCTGVYDRELLARLDRVCEDCYNIYRDVGVAAECRSDCFHNEVFLYCVDYMYRPRQRNQ
YRAALQRLGK
>ROT60582.1 CHH 3 precursor [Penaeus vannamei]

MVAVQLAVLMCMLLAVPAAITTHDNTNELPKFLLSSPGDSLTSGQSLIKRTTSFSSCTGVYD
RELLARLDRVCEDCYNLYRDVGVAAECRSNCFHNEVFLYCVDYMYRPRQRNQYRAALQ
RLGK
>ROT60584.1 CHH 1 precursor [Penaeus vannamei]
MQDADDRLYKATSTWLRRRADEEPRSRRFSPPDSSRRRGNDWGSTAVLVSLLLVFPASVL
ASWDGNEIPPSLPSSSESSPATSLAGAQTANKRSISFDSCTGVYDRELLVRLDRVCEDCYNL
YRNTDVAVECRSNCFHNEVFLYCVDYMYRPRQRNQYRAALQRLGK
>ROT60585.1 CHH 2 precursor [Penaeus vannamei]
MFASRMVWSALVLSLMVALAASAATWDRSFRDEEEPPKFLPPSSPDSSAVALPRTLPLPAD
EDHSLSKRSGYYNSCTGVYDRELIARLDRVCEDCYNLYRDVEVAVGCRKGCYHNEVFLY
CVDYMFRPRQRNQYRAALQKLGK
>ROT60586.1 hypothetical protein C7M84_011789 [Penaeus vannamei]
MIGVRLVRSAVLVSLLLVFPASVLASWDGNEIPPSLPSSSESSPATSLAGAQTANKRSISFDS
CTGVYDRELLVRLDRVCEDCYNLYRDTDVAVECRSNCFHNEVFLYCVDYMYRPRQRNQY
RAALQRLGK
>ROT60587.1 hypothetical protein C7M84_011790 [Penaeus vannamei]
MIGVRLVRSAVLVSLLLVFPASVLASWDGNEIPPSLPSSSESSPATSLAGAQTANKRSISFDS
CTGVYDRELLVRLDRVCEDCYNLYRDTDVAVECRSNCFHNEVFLYCVDYMYRPRQRNQY
RAALQRLGK
>ROT60588.1 hypothetical protein C7M84_011791 [Penaeus vannamei]
MIGVRLVRSAVLVSLLLVFPASVLASWDGNEIPPSLPSSSESSPATSLAGAQTANKRSISFDS
CTGVYDRELLVRLDRVCEDCYNLYRDTDVAVECRSNCFHNEVFLYCVDYMYRPRQRNQY
RAALQRLGK
>ROT60589.1 hypothetical protein C7M84_011793 [Penaeus vannamei]
MIGVRLVRSAVLVSLLLVFPASVLASWDGNEIPPSLPSSSESSPATSLAGAQTANKRSISFDS
CTGVYDRELLVRLDRVCEDCYNLYRDTDVAVECRSNCFHNEVFLYCVDYMYRPRQRNQY
RAALQRLGK
>ROT60590.1 CHH 3 precursor [Penaeus vannamei]
MGFLQRRPLTAVPKPENMHSRHQTLTTAYIRRRPLGSVAEQTKSLEVAGSLLPIRVDAEEMI
GVRLVRSAVLVSLLLVFPASVLASWDGNEIPPSLPSSSESSPATSLAGAQTANKRSISFDSCT
GVYDRELLVRLDRVCEDCYNLYRDTDVAVECRSNCFHNEVFLYCVDYMYRPRQRNQYR
AALQRLGK
>ROT60591.1 CHH 3 precursor [Penaeus vannamei]
MILQTTKPEADSLGIYLVCCRQHRADIRTPAGPHITKHAHHPKTSLHQAEFLAKRIHHHSH
AAGNNNIPLTGRSMTLLAAHHFKSPRQPCSSPASPRLAPDPKYRPPPPPARTLPPPEDALPL
APPAHSVTLPEDGIPSAPPAHCSSEAREHALSASRFRDQRLRTQGVSPKPLISPTCRTLTTAYI
RRRPLGSVAEQTKSLEVAGSLLPIRVDAEEMIGVRLVRSAVLVSLLLVFPASVLASWDGNEI
PPSLPSSSESSPATSLAGAQTANKRSISFDSCTGVYDRELLVRLDRVCEDCYNLYRDTDVAV
ECRSNCFHNEVFLYCVDYMYRPRQRNQYRAALQRLGK
>ROT60592.1 CHH 1 precursor [Penaeus vannamei]
MKDADDRLYKATSTWLRRRADEEPRSRRFSPPDSSRRRGNDWGSTGAFSCPGIPAASVPA
SVLASWDGNEIPPSLPSSSESSPATSLAGAQTANKRSISFDSCTGVYDRELLVRLDRVCEDC
YNLYRDTDVASNAEQTKSLEVAGSLLPIRVDAEEMIGVRLVRSAVLVSLLLVFPASVLASW
DGNEIPPSLPSSSESSPATSSREPRPQTSAAYPSTRARASTTANSLSNCFHNEVFLYCVDYMY

RPRQRNQYRAALQRLGK
>ROT60593.1 CHH 3 precursor [Penaeus vannamei]
MYLSAPPPARTLPPPEDALPSAPPAHSVTPPEDGILQRRPLTAVPKPENMHSRHQTLTTAYIR
RRPLGSVAEQTKSLEVAGSLLPIRVDAEEMIGVRLVRSAVLVSLLLVFPASVLASWDGNEIP
PSLPSSSESSPATSLAGAQTANKRSISFDSCTGVYDRELLVRLDRVCEDCYNLYRDTDVAVE
CRSNCFHNEVFLYCVDYMYRPRQRNQYRAALQRLGK
>ROT60594.1 CHH 3 precursor [Penaeus vannamei]
MQDADDRLYKATSTWLRRRADEEPRSRRFSPPDSSRREEMIGVRLCFPASVLASWDGNEI
LRPCLPPQNPLLRPPSGAQTANKRSISFDSCTGVYDRELLVRLDRVCEDCYNLYRDTDVAV
ECRSNCFHNEVFLYCVDYMYRPRQRNQYRAALQRLGK
>ROT60595.1 CHH 2 precursor [Penaeus vannamei]
MILQTTKPEADTVKANLYNASASTPYPAKHTVTQSQLLTPQHLSRLLPPHRAISGQNITKD
ADHPKTSLHRAEFLAKRIHHRSHAAGSNNTPLTGRAMTLLATHCFKSTRQPAAPSFSSLAP
HQNPTLTTAYIRRRPLGSVAEQTKSLEVAGSLLPIRVDAEEMIGVRLVRSAVLVSLLLVFPAS
VLASWDGNEIPPSLPSSSESSPATSLAGAQTANKRSISFDSCTGVYDRELLVRLDRVCEDCY
NLYRDTDVAVECRSNCFHNEVFLYCVDYMYRPRQRNQYRPPCRGSASRRFLSGHTFYGD
ARAMISRRPSQRRTVLGRRGGGDRAISPSVLPGNVLKI
>ROT60596.1 CHH 1 precursor [Penaeus vannamei]
MKDADDRLYKATSTWLRRRADEEPRSRRFSPPDSSRRRGNDWGSTVFPASVLASWDGNE
IPPSLPSSSESSPATSLAGAQTANKRSISFDSCTGVYDRELLVRLDRVCEDCYNLYRDTDVAV
ECRSNCFHNEVFLYCVDYMYRPRQRNQYRAALQRLGK
>ROT61446.1 hypothetical protein C7M84_020778 [Penaeus vannamei]
MDHNKIVLVSASILVLITVLVSHNSNGVKFVLKLNTSCNMSDRLSIRRKRQTFDASCKGVY
DRRLWTKLNRVCLDCQNIYRKDTTIERDCRKNCFGTEIFYGCILTLNLPKKYYLFYADLLR
E
>ROT63548.1 CHH0 [Penaeus vannamei]
MDNKIAFVSASVLLLVAVLASHNGVHARSVVPEGLQELEIPRQESDMFAVRRKRQVFDAS
CKGVYDRGLWAKLNNACLDCQNIYRGNPAIEGECRQNCFGTEVFYGCIKALKLPTKNYL
YFAEVLRES
>ROT65053.1 hypothetical protein C7M84_017001 [Penaeus vannamei]
MFGMQQNRSLNSVGRSRTVLLLVALLLCQESSASFIKLRPNTLREFQFLQCRGDYDKERYT
ALSRVCDDCHNLFRQPQVMTDCKSNCFRNSFFLTCVNLLKLEHLEDDFKNNIMIVSGNEL
>ROT66419.1 CHH-like peptide precursor [Penaeus vannamei]
MWVILALTASCSVMCHARIMDPGHSRPPFHPLSPPSPSTSSSSASALRVAKRDVFDPSCKGI
YNRAIWAKLNRACEDCQNLFRDEMGIYESCREKCFDTKIFPACVIELSLNLNEYMFEAELI
REL
>ROT66421.1 putative thioredoxin domain-containing protein 16-like isoform X1 [Penaeus vannamei]
MWVILALTASCSVMCHARIMDPGHSRPPFHPPSPPSPSTSSSSASALRVAKRDVFDPSCKGI
YNRAIWAKLNRACEDCQNLFRDEMGIYESCREKCFDTKIFPACVIELSLNLNEYMFEAELI
REL
>ROT66425.1 CHH-like peptide precursor [Penaeus vannamei]
MWVILALTASCSVMCHARIMDPGHSRPPFHPPSPPSPSTSSSSASALRVAKRDVFDPSCKGI
YNRAIWAKLNRACEDCQNLFRDEMGIYESCREKCFDTKIFPACVIELSLNLNEYMFEAELI

REL
>ROT66427.1 hypothetical protein C7M84_015562 [Penaeus vannamei]
MWVILALTASCSVMCHARIMDPGHSRPPFHPLSPPSPSTSSSSASALRVAKRDVFDPSCKGI
YNRAIWAKLNRACEDCQNLFRDEMGIYESCREKCFDTKIFPACVIELSLNLNEYMFEAELI
REL
>ROT66630.1 CHH precursor [Penaeus vannamei]
MCFSSKMAVALVVVVACSTTWARSAEESSSPVASLIRGRSLSKRANFDPSCTGVYDRELLG
RLSRLCDDCYNVFREPKVATECRSNCFYNPVFVQCLEYLIPADLHEEYQALVQTVGK
>ROT67683.1 hypothetical protein C7M84_014214 [Penaeus vannamei]
MDHNKIVLVSASILVLITVLVSHNSNGVKFVLKLNTSCNMSDRLSIRRKRQTFDASCKGVY
DRRLWTKLNRLCLDCQNIYRKDTTIERDCRKNCFGTEIFYGCILTLNLPKKYYLFYADLLR
E
>ROT67920.1 ITP [Penaeus vannamei]
MARKHPCHGDKISPQHPPTAFAFRRAVAEADFAGSLQRQLERKMVVPKALHFDKIFQKRL
MLFLVLMICQQGYASFIKVRPNTLREFQFLKCQGEFNKAQYVSLSHVCEDCHNLYRQPEIL
TECKANCFQNTLFPTCVSLLMLDRHEDDLNKKVALISGQEL
>ROT68737.1 CHHs 3 [Penaeus vannamei]
MIALRLMAVTLLVALAASTTWARSFNKRANFDPSCTGVYDRELLGRLSRLCDDCYNVFRE
PKVATECRSNCFYNPVFVQCLEYLIPADLHEEYQALVQTVGK
>ROT68738.1 CHHs 5 [Penaeus vannamei]
MWSAAIVTLLVAAAACASSWERSLEMEGQTSEFLPSFPQSPSLLSSAADHSLRKRSIFDHS
CTGVFDRELIGRLNRVCDDCYNVFRDTDVATGCRSNCFYNRMFLQCLVYLFPPRFRNQYK
AAVQMVGKARRWFVEPDAGSRIFFDDFRRQMDQPHRLYIKGTAAFWSQSNCLRGRSHSR
QLLIL
>ROT68740.1 CHH0-like peptide precursor [Penaeus vannamei]
MVSFLSLRMVCSAALVSLLVLALSSRSAFARSVDGVGRLEKLLSSSSSSSGSSSPLDALGG
DHSVNKRDTFDHSCKGIYDRELFRKLDRVCEDCYNLYRKPYVATECKSNCFVNKRFNVCV
ADLRHDVSRFLKMAKFLRYP
>ROT71349.1 hypothetical protein C7M84_010321 [Penaeus vannamei]
MDHNKIVLVSASILVLITVLVSHNSNGVKFVLKLNTSCNMSDRLSIRRKRQTFDASCKGVY
DRRLWTKLNRVCLDCQNIYRKDTTIERDCRKNCFGTEIFYGCILTLNLPKKYYLFYADLLR
E
>ROT78345.1 hypothetical protein C7M84_002930 [Penaeus vannamei]
MDHNKIVLVSASILVLITVLVSHNSNGVKFVLKLNTSCNMSDRLSIRRKRQTFDASCKGVY
DRRLWTKLNRVCLDCQNIYRKDTTIERDCRKNCFGTEIFYGCILTLNLPKKYYLFYADLLR
E
>ROT78398.1 CHHs 1 [Penaeus vannamei]
MMGWRSAVSAQTDTQNHHISSAKRCSIFCANYACLPYYVVSDNGLFAAAARGVVRCPRR
RLIRPYHGLTKRSLFDPSCTGVFDRQLLRRLRRVCDDCFNVFREPNVAIDCRENCYNNEVF
RQCMAYVVPANLHDEHRQAVQMVGK
>ROT78399.1 CHH0 [Penaeus vannamei]
MTAFRMVWSMLLASLLLLLAASSAAPADALSAPAAGLTKRSLFDPSCTGVFDRQLLRRLR
RVCDDCFNVFREPNVSTECRSNCYNNEVFRQCMEYLLPPHLHEEHRLAVQMVGK
>ROT79881.1 hypothetical protein C7M84_001399 [Penaeus vannamei]

MDHNKIVLVSASILVLITVLVSHNSNGVKFVLKLNTSCNMSDRLSIRRKRQTFDASCKGVY
DRRLWTKLNRVCLDCQNIYRKDTTIERDCRKNCFGTEIFYGCILTLNLPKKYYLFYADLLR
E
>ROT79883.1 hypothetical protein C7M84_001401 [Penaeus vannamei]
MDHNKIVLVSASILVLITVLVSHNSNGVKFVLKLNTSCNMSDRLSIRRKRQTFDASCKGVY
DRRLWTKLNRVCLDCQNIYRKDTTIERDCRKNCFGTEIFYGCILTLNLPKKYYLFYADLLR
E
>ROT81026.1 MIH1 [Penaeus vannamei]
MQRILYKGGSRPTTALHSRRLRTPLSAPSSSAFVHLYAYTLMYRLAMRTWLVVILVVVGTS
LFFDTASASLIHGTCRGVMGNREIYEKVVRVCEDCTNIFRMPGLDGMCRDRCFYNEWFLL
CLKAANREDEIENFRVWISILNAGQ
>ROT81027.1 putative MIH [Penaeus vannamei]
MRTWLALVIVLVGTSLFVDTASASFTDGACRGIMGNREIYKKVERVCEDCTNIFRLPGLDG
MCRDRCFYNEWFLLCLKAANREDEIENFRVWISILNA
>ROT81028.1 MIH1 [Penaeus vannamei]
MYRPVIRIWLALVIAVVGASIFFDSASASFIDGSCRGVMGNREIYKKVVRVCEDCTNIFRLP
GLDVMCRDRCFHNEWFLLCLNAANREDEIENFKVWISILSAGQ
>ROT81029.1 hypothetical protein C7M84_000234 [Penaeus vannamei]
MYHLAIRTWLAIVLVMVGISLFFDTASALFMDGSCRGLMGNRDIYKKVVRVCEDCTNIFR
LPGLDGLCRNRCFYNEWFLVCLKAANREGEIENFRVWVSILSA
>ROT81030.1 neuropeptide Pem-SGP-C2 precursor [Penaeus vannamei]
MGGTCPGRMGNREMYTKVDRVCEDCANIFRLPVLEGLCRDRCFYNEWFLLCLKAANRE
DEIENFRVWISILNA
>ROT81031.1 MIH2 [Penaeus vannamei]
MYRLAIRSWLPVMTVLFATSLFFDTASASPIDGTCPGRMGNREIYKKVDSVCKDCVNIFRL
PELEGLCRDECFINDWFLFCAKAAKRMDEIENFRVWISILNA
>ROT85393.1 hypothetical protein C7M84_015242 [Penaeus vannamei]
MDHNKIVLVSASILVLITVLVSHNSNGVKFVLKLNTSCNMSDRLSIRRKRQTFDASCKGVY
DRRLWTKLNRVCLDCQNIYRKDTTIERDCRKNCFGTEIFYGCILTLNLPKKYYLFYADLLR
E
>XP_027221932.1 CHHs-like [Penaeus vannamei]
MFASRMVWSALVLSLMVALAASAATWDRSFRDEEEPPKFLPPSSPDSSAVALPRTLPLPAD
EDHSLSKRSGYYNSCTGVYDRELIARLDRVCEDCYNLYRDVEVAVGCRKGCYHNEVFLY
CVDYMFRPRQRNQYRAALQKLGK
>XP_027223579.1 CHH [Penaeus vannamei]
MIALRLMAVTLLVALAASTTWARSFNKRANFDPSCTGVYDRELLGRLSRLCDDCYNVFRE
PKVATECRSNCFYNPVFVQCLEYLIPADLHEEYQALVQTVGK
>XP_027231748.1 CHH 6-like [Penaeus vannamei]
MDNKIAFVSASVLLLVAVLASHNGVHARSVVPEGLQELEIPRQESDMFAVRRKRQVFDAS
CKGVYDRGLWAKLNNACLDCQNIYRGNPAIEGECRQNCFGTEVFYGCIKALKLPTKNYL
YFAEVLRES

Amino acid sequences of 52 CHH family member proteins from the genome of Litopenaeus vannamei

>LVANscaffold_1179_1
MDHNKIVLVSASILVLITVLVSHNSNGVKFVLKLNTSCNMSDRLSIRRKRQTFDASCKGVY
DRRLWTKLNRVCLDCQNIYRKDTTIERDCRKNCFGTEIFYGCILTLNLPKKYYLFYADLLR
E
>LVANscaffold_1179_2
MDHNKIVLVSASILVLITVLVSHNSNGVKFVLKLNTSCNMSDRLSIRRKRQTFDASCKGVY
DRRLWTKLNRVCLDCQNIYRKDTTIERDCRKNCFGTEIFYGCILTLNLPKKYYLFYADLLR
E
>LVANscaffold_1399
MDHNKIVLVSASILVLITVLVSHNSNGVKFVLKLNTSCNMSDRLSIRRKRQTFDASCKGVY
DRRLWTKLNRVCLDCQNIYRKDTTIERDCRKNCFGTEIFYGCILTLNLPKKYYLFYADLLR
E
>LVANscaffold_2306
MDHNKIVLVSASILVLITVLVSHNSNGVKFVLKLNTSCNMSDRLSIRRKRQTFDASCKGVY
DRRLWTKLNRVCLDCQNIYRKDTTIERDCRKNCFGTEIFYGCILTLNLPKKYYLFYADLLR
E
>LVANscaffold_2780
MDHNKIVLVSASILVLITVLVSHNSNGVKFVLKLNTSCNMSDRLSIRRKRQTFDASCKGVY
DRRLWTKLNRLCLDCQNIYRKDTTIERDCRKNCFGTEIFYGCILTLNLPKKYYLFYADLLR
E
>LVANscaffold_290_1
MDHNKIVLVSASILVLITVLVSHNSNGVKFVLKLNTSCNMSDRLSIRRKRQTFDASCKGVY
DRRLWTKLNRVCLDCQNIYRKDTTIERDCRKNCFGTEIFYGCILTLNLPKKYYLFYADLLR
E
>LVANscaffold_290_2
MDHNKIVLVSASILVLITVLVSHNSNGVKFVLKLNTSCNMSDRLSIRRKRQTFDASCKGVY
DRRLWTKLNRVCLDCQNIYRKDTTIERDCRKNCFGTEIFYGCILTLNLPKKYYLFYADLLR
E
>LVANscaffold_3666
MDHNKIVLVSASILVLITVLVSHNSNGVKFVLKLNTSCNMSDRLSIRRKRQTFDASCKGVY
DRRLWTKLNRVCLDCQNIYRKDTTIERDCRKNCFGTEIFYGCILTLNLPKKYYLFYADLLR
E
>LVANscaffold_4017_1
MDHNKIVLVSASILVLITVLVSHNSNGVKFVLKLNTSCNMSDRLSIRRKRQTFDASCKGVY
DRRLWTKLNRLCLDCQNIYRKDTTIERDCRKNCFGTEIFYGCILTLNLPKKYYLFYADLLR
E
>LVANscaffold_4017_2
MDHNKIVLVSASILVLITVLVSHNSNGVKFVLKLNTSCNMSDRLSIRRKRQTFDASCKGVY
DRRLWTKLNRVCLDCQNIYRKDTTIERDCRKNCFGTEIFYGCILTLNLPKKYYLFYADLLR
E
>LVANscaffold_4164
MDHNKIVLVSASILVLITVLVSHNSNGVKFVLKLNTSCNMSDRLSIRRKRQTFDASCKGVY
DRRLWTKLNRVCLDCQNIYRKDTTIERDCRKNCFGTEIFYGCILTLNLPKKYYLFYADLLR
E

>LVANscaffold_4572_1
MDHNKIVLVSASILVLITVLVSHNSNGVKFVLKLNTSCNMSDRLSIRRKRQTFDASCKGVY
DRRLWTKLNRVCLDCQNIYRKDTTIERDCRKNCFGTEIFYGCILTLNLPKKYYLFYADLLR
E
>LVANscaffold_4572_2
MDHNKIVLVSASILVLITVLVSHNSNGVKFVLKLNTSCNMSDRLSIRRKRQTFDASCKGVY
DRRLWTKLNRVCLDCQNIYRKDTTIERDCRKNCFGTEIFYGCILTLNLPKKYYLFYADLLR
E
>LVANscaffold_4572_3
MDHNKIVLVSASILVLITVLVSHNSNGVKFVLKLNTSCNMSDRLSIRRKRQTFDASCKGVY
DRRLWTKLNRVCLDCQNIYRKDTTIERDCRNILLLYRNILWVHLDLNLPKKYYLFYADLL
RE
>LVANscaffold_2490_1
VRSAVLVSLLLVFPASVLASWDGNEIPPSLPSSSESSPATSLAGAQTANKRSISFDSCTGVYD
RELLVRLDRVCEDCYNLYRDTDVAVECRSNCFHNEVFLYCVDYMYRPRQRNQYRAALQR
LGK
>LVANscaffold_2490_3
MIGVRLVRSAVLVSLLLVFPASVLASWDGNEIPPSLPSSSESSPATSLAGAQTANKRSISFDS
CTGVYDRELLVRLDRVCEDCYNLYRDTDVAVECRSNCFHNEVFLYCVDYMYRPRQRNQY
RAALQRLGK
>LVANscaffold_2490_4
MRAAVLVSLLVAIPASATTSGDENEIPTLLRHTQKVSPVSSFAGAHSLHKRSLSFRSCTGVY
DRELLARLDRVCEDCYNIYRDVGVAAECRSDCFHNEVFLYCVDYMYRPRQRNQYRAAL
QRLGK
>LVANscaffold_2490_5
MVAVQLAVLMCMLLAVPAAITTHDNTNELPKFLLSSPGDSLTSGQSLIKRTTSFSSCTGVYD
RELLARLDRVCEDCYNLYRDVGVAAECRSNCFHNEVFLYCVDYMYRPRQRNQYRAALQ
RLGK
>LVANscaffold_2490_6
MIGVRLVRSAVLVSLLLVFPASVLASWDGNEIPPSLPSSSESSPATSLAGAQTANKRSISFDS
CTGVYDRELLVRLDRVCEDCYNLYRDTDVAVECRSNCFHNEVFLYCVDYMYRPRQRNQY
RAALQRLGK
>LVANscaffold_2490_7
MIGVRLVRSAVLVSLLLVFPASVLASWDGNEIPPSLPSSSESSPATSLAGAQTANKRSISFDS
CTGVYDRELLVRLDRVCEDCYNLYRNTDVAVECRSNCFHNEVFLYCVDYMYRPRQRNQY
RAALQRLGK
>LVANscaffold_2490_10
MIGVRLVRSAVLVSLLLVFPASVLASWDGNEIPPSLPSSSESSPATSLAGAQTANKRSISFDS
CTGVYDRELLVRLDRVCEDCYNLYRDTDVAVECRSNCFHNEVFLYCVDYMYRPRQRNQY
RAALQRLGK
>LVANscaffold_2490_11
MIGVRLVRSAVLVSLLLVFPASVLASWDGNEIPPSLPSSSESSPATSLAGAQTANKRSISFDS
CTGVYDRELLVRLDRVCEDCYNLYRDTDVAVECRSNCFHNEVFLYCVDYMYRPRQRNQY
RAALQRLGK

>LVANscaffold_2490_12
MIGVRLVRSAVLVSLLLVFPASVLASWDGNEIPPSLPSSSESSPATSLAGAQTANKRSISFDS
CTGVYDRELLVRLDRVCEDCYNLYRDTDVAVECRSNCFHNEVFLYCVDYMYRPRQRNQY
RAALQRLGK
>LVANscaffold_2490_14
MIGVRLVRSAVLVSLLLVFPASVLASWDGNEIPPSLPSSSESSPATSLAGAQTANKRSISFDS
CTGVYDRELLVRLDRVCEDCYNLYRDTDVAVECRSNCFHNEVFLYCVDYMYRPRQRNQY
RAALQRLGK
>LVANscaffold_2490_15
MIGVRLVRSAVLVSLLLVFPASVLASWDGNEIPPSLPSSSESSPATSLAGAQTANKRSISFDS
CTGVYDRELLVRLDRVCEDCYNLYRDTDVAVECRSNCFHNEVFLYCVDYMYRPRQRNQY
RAALQRLGK
>LVANscaffold_2490_16
MIGVRLVRSAVLVSLLLVFPASVLASWDGNEIPPSLPSSSESSPATSLAGAQTANKRSISFDS
CTGVYDRELLVRLDRVCEDCYNLYRDTDVAVECRSNCFHNEVFLYCVDYMYRPRQRNQY
RAALQRLGK
>LVANscaffold_2490_17
MIGVRLVRSAVLVSLLLVFPASVLASWDGNEIPPSLPSSSESSPATSLAGAQTANKRSISFDS
CTGVYDRELLVRLDRVCEDCYNLYRDTDVAVECRSNCFHNEVFLYCVDYMYRPRQRNQY
RAALQRLGK
>LVANscaffold_2490_20
MIGVRLVRSAVLVSLLLVFPASVLASWDGNEIPPSLPSSSESSPATSLAGAQTANKRSISFDS
CTGVYDRELLVRLDRVCEDCYNLYRDTDVAVECRSNCFHNEVFLYCVDYMYRPRQRNQY
RAALQRLGK
>LVANscaffold_2490_22
MIGVRLVRSAVLVSLLLVFPASVLASWDGNEIPPSLPSSSESSPATSLAGAQTANKRSISFDS
CTGVYDRELLVRLDRVCEDCYNLYRDTDVAVECRSNCFHNEVFLYCVDYMYRPRQRNQY
RPPCRGSAS
>LVANscaffold_2490_23
MIGVRLVRSAVLVSLLLVFPASVLASWDGNEIPPSLPSSSESSPATSLAGAQTANKRSISFDS
CTGVYDRELLVRLDRVCEDCYNLYRDTDVAVECRSNCFHNEVFLYCVDYMYRPRQRNQY
RAALQRLGK
>LVANscaffold_1388_1
MTAFRMVWSMLLASLLLLLAASSAAPADALSAPAAGLTKRSLFDPSCTGVFDRQLLRRLR
RVCDDCFNVFREPNVSTECRSNCYNNEVFRQCMEYLLPPHLHEEHRLAVQMVGK
>LVANscaffold_1388_2MTAFRMVVSDNGLFAAAARGVVRCPRRRLIRPYHGLTKRSLFDPS
CTGVFDRQLLRRLRRVCDDCFNVFREPNVAIDCRENCYNNEVFRQCMAYVVPANLHDEH
RQAVQMVGK
>LVANscaffold_2640_1
MAVTLLVALAASTTWARSFNKRANFDPSCTGVYDRELLGRLSRLCDDCYNVFREPKVATE
CRSNCFYNPVFVQCLEYLIPADLHEEYQALVQTVGK
>LVANscaffold_2640_2
MWSAAIVTLLVAAAACASSWERSLEMEGQTSEFLPSFPQSPSLLSSAADHSLRKRSIFDHS
CTGVFDRELIGRLNRVCDDCYNVFRDTDVATGCRSNCFYNRMFLQCLVYLFPPRFRNQYK

AAVQMVGK
>LVANscaffold_2640_3
MTAFRLMAVALVVVVACSTTWARSAEESSSPVASLIRGRSLSKRANFDPSCTGVYDRELLG
RLSRLCDDCYNVFREPKVATECRSNCFYNPVFVQCLEYLIPADLHEEYQALVQTVGK
>LVANscaffold_2640_4
MVSFLSLRMVCSAALVSLLVLALSSRSAFARSVDGVGRLEKLLSSSSSSSGSSSPLDALGG
DHSVNKRDTFDHSCKGIYDRELFRKLDRVCEDCYNLYRKPYVATECKSNCFVNKRFNVCV
ADLRHDVSRFLKMAKFLRYP
>LVANscaffold_2739
MVVPKALHFDKIFQKRLMLFLVLMICQQGYASFIKVRPNTLREFQFLKCQGEFNKAQYVS
LSHVCEDCHNLYRQPEILTECKANCFQNTLFPTCVSLLMLDRHEDDLNKKVALISGQEL
>LVANscaffold_2916
MTAFRLMAVALVVVVACSTTWARSAEESSSPVASLIRGRSLSKRANFDPSCTGVYDRELLG
RLSRLCDDCYNVFREPKVATECRSNCFYNPVFVQCLEYLIPADLHEEYQALVQTVGK
>LVANscaffold_2938_2
MWVILALTASCSVMCHARIMDPGHSRPPFHPLSPPSPSTSSSSASALRVAKRDVFDPSCKGI
YNRAIWAKLNRACEDCQNLFRDEMGIYESCREKCFDTKIFPACVIELSLNLNEYMFEAELI
REL
>LVANscaffold_2938_3
MWVILALTASCSVMCHARIMDPGHSRPPFHPPSPPSPSTSSSSASALRVAKRDVFDPSCKGI
YNRAIWAKLNRACEDCQNLFRDEMGIYESCREKCFDTKIFPACVIELSLNLNEYMFEAELI
REL
>LVANscaffold_2938_4
MWVILALTASCSVMCHARIMDPGHSRPPFHPPSPPSPSTSSSSASALRVAKRDVFDPSCKGI
YNRAIWAKLNRACEDCQNLFRDEMGIYESCREKCFDTKIFPACVIELSLNLNEYMFEAELI
REL
>LVANscaffold_3152
MFGMQQNRSLNSVGRSRTVLLLVALLLCQESSASFIKLRPNTLREFQFLQCRGDYDKERYT
ALSRVCDDCHNLFRQPQVMTDCKSNCFRNSFFLTCVNLLKLEHLEDDFKNNIMIVSGNEL
>LVANscaffold_3427
MDNKIAFVSASVLLLVAVLASHNGVHARSVVPEGLQELEIPRQESDMFAVRRKRQVFDAS
CKGVYDRGLWAKLNNACLDCQNIYRGNPAIEGECRQNCFGTEVFYGCIKALKLPTKNYL
YFAEVLRES
>LVANscaffold_1036_1
MYRLAMRTWLVVILVVVGTSLFFDTASASLIHGTCRGVMGNREIYEKVVRVCEDCTNIFR
MPGLDGMCRDRCFYNEWFLLCLKAANREDEIENFRVWISILNAGQ
>LVANscaffold_1036_2
MRTWLALVIVLVGTSLFVDTASASFTDGACRGIMGNREIYKKVERVCEDCTNIFRLPGLDG
MCRDRCFYNEWFLLCLKAANREDEIENFRVWISILNA
>LVANscaffold_1036_3
MYRPVIRIWLALVIAVVGASIFFDSASASFIDGSCRGVMGNREIYKKVVRVCEDCTNIFRLP
GLDVMCRDRCFHNEWFLLCLNAANREDEIENFKVWISILSAGQ
>LVANscaffold_1036_4
MYHLAIRTWLAIVLVMVGISLFFDTASALFMDGSCRGLMGNRDIYKKVVRVCEDCTNIFR

LPGLDGLCRNRCFYNEWFLVCLKAANREGEIENFRVWVSILSA
>LVANscaffold_1036_5
MYRLATRTWLAIVIVLYATSFFFGTASASLMGGTCPGRMGNREMYTKVDRVCEDCANIFR
LPVLEGLCRDRCFYNEWFLLCLKAANREDEIENFRVWISILNA
>LVANscaffold_1036_6
MYRLAIRSWLPVMTVLFATSLFFDTASASPIDGTCPGRMGNREIYKKVDSVCKDCVNIFRL
PELEGLCRDECFINDWFLFCAKAAKRMDEIENFRVWISILNA
>VIH1028_1
MRTWLLLAIVAVGASLANILDSNCRGAMGNRDMYRKVERVCEDCTNIYRLPQLDGLCRN
RCFNNQWFLMCLHSAKREAELGHFRLWISILNAGRPW
>VIH1028_2
MWAVPSSRQGSGHFSPKATRLMASPRRTPSILKKACQVALVAAVLYGLLTAPASARFIDDEC
VGAMGNRNIYEKVARVCDDCSNIFRLPNVGESCRRNCFYNEDFLWCIMASERHAEVEQF
NRWISILKAGRK
>MIH1028
MYRLAMKTWLAIVIVVVGTSLFFDTTSASFIDGTCRGVMGNRDIYKKVVRVCEDCTNIFR
LPGLDGMCRDRCFYNEWFLICLKAANREDEIEKFKVWISILNAGQ
