# Supplementary material for: Identification and Functional Analysis of a Key Gene in the CHH Gene Family for Glucose Metabolism in the Pacific White Shrimp Litopenaeus vannamei
Source: Int J Mol Sci. 2025 May 12;26(10):4612. doi: 10.3390/ijms26104612 (PMC12111282; doi:10.3390/ijms26104612)
Supplement: Supplementary file 1 [file ijms-26-04612-s001.zip › Date S3-LvCHH Ia sequences.pdf]

Sequence of *LvCHH Ia* gene

>NC_091563.1:17786966-17792732 *Penaeus vannamei* isolate JL-2024 chromosome 15, ASM4276789v1, whole genome shotgun sequence

AGACAGTTCGGCGAAGCATCCCCGTTAACAGTAGAGCCAAAGCCCTTGTTCGGTCTCTCTCGCTCGCGCTCT
TGCTCTCAGACCAGCATCCCGAGTGAGAACGCAATTACAGGTGTACTACCTTGTGTGTTATCGTTGCAATATC
CACCTGCATTTGCAACCGCAACCATGGTCAGCTTCCTCTCACTTCGCATGGTAAGTGATGTGTATATATATGGC
TGTTCGTTGCTGTGTTTCTTGGTGTTGATGGATTTGCGTTGGTTTCTTTTCTTTGTCTTTTTTATTGTGTATGTG
AATTAGATTTTTATTGTGAATTGTCCGTAGCTCAATGGTAGCGACTCGATTCGCTATCAACAAGTGTCCGTTAC
TCTAGCTCTTGTCCCTCGCTCATTCCACACGCGCCCCCTTTATCAAAACGCTCTCAAAATCAAACTCAAACTAA
CTAAAATCAAGAACAAAATTCAGGAAGCGCGAGTCACTGTTAAGATAGGCTTCTTTATTCCTTAGTTTACCGT
CAGTGTACAAAGAAAAGACAAAAAGAAATCTTATGCATCCGGTACATAAAGCTCTCCCTCCGGGCGACAAA
GTCTGTCTAAATTCAAAGCGAGTGTAATGTGCCCCATAATGAAAAGGATACGATAGTTTTTTTCTTCTTATCTG
GAAAAAAGACTCAGGATATCCTACAGCCATGCTATTTATTATTTATGAAGAATTCATGTTTCCCTTTTACTTTCT
TTCTTTCATACATATTTTTTCTTTCTCTAGAAAGCGTTTGCACAAAGGTTAGATTTTTATATTGACACAGTTGAA
TCGTCTGAGTACAATCGAACTAAAATAAATATTTGTTTATTTATTCTGATGCATGGAAATCATATCAAAATAGAT
GACAGGTAGATTAAAAATAATCAAACCAAAAACACTTAGTGCAATAATCTTTACACTAGTCTGGCTCCAGCAA
CACAACCGTGATCCCAGTTACCACCAGGGAATCAGTCACCCTTCTTAGAAATTCCCCGTGGGCGCCTCAGGG
CCTCTTCAGACTCCTCGAAGGGCTCATGGCAACCCCGCGCGGATATGTAGTTCTTTGTAGTTTCCTCAACAGC
ATGATGTGTATGGCATGCAAAGGCTCATACTCATGCACATACATAAACTCTTGTATGTTGTGTTCATAGAGAAT
CTATCTGTCTATCTATATCTGTATCTATACATATACATTCATTCATATACATATATATATATATATATATATATATAT
ATATATATATATACATATATGTATATATATATACATATATATATATACATATATGTATATATATACATATATATATACATATAT
ATATACATATATATATACATATATATATATATATATACATATATATATATATATACATATATATACATATATATATATATACAT
ATATGTATATATATATATATATATATATATATATATATATATATATATATATATATCGATAGTATAGGAATGTGTGTATGTATA
GATATATGTATTCATTTATGTCATCTTAAATCATATTACTTTTTATAGATTAATCTATTTAATGGGATTGGAGGATT
TCTCGGGTTTTCTCTAATTCGTTCTGATTCTCTCTCTCTCTCTCTCTCTCTCTCTCTCTCTCTCTCTCTCTCTCT
CTCTCTCTCTCTCTCTCTCTCTCTCTCTCTCTCTCTCTCTCTCTCTTTCTCTCTATTTACCTCTATCTATCTATCTA
TCCATCTATCTATCTACCTCTCTCTCCCTCTCTCTCTCATTCTCTTTCTTTTTACTCATTCTTTCCCGTTTCTTCCCA
CTTCGTCGACTTCGTCTCTCTCTTCCGCCTTCTTGTTTTCCATCTGTGTCCCCAGCTTCCTCATCCGCGCTCACT
GTCTATCACTGCAGATTGCCTTCTTCCTCTGATGTTTAGACTCTGTAATGCATTCATCAATCATCCCACAAGAG
AAAAAAAATATGTATATGTACTTGCGCAGGTGAGAAGGGGGATTAGGGAAGGGGCGGGGGGTAGGAGGG
GGGGCAAGAGGGGGGGGGGGAAGAAAGCGCGTGAATGGAGGGGATAAGTGAGTGCGCGATGCGTCACC
TGAGGGCTTATTTGTTCACCGGATATGTGGCTATGCTATCGCGTGTCAACCGGTTTGCTTCACCGGGCACCTG
GCAACAGACTCATCCCAGAAGCCGGGCTTTTTTTTTCTTTTTTTCTTTTTTTCTTTTTTTTTTTTTTGTTATTTTA
TTTATCTTTTCTTTTTTTTTTCTTTTTTTTGATTGCTGATCGTGTGGGTCACTCATCAATTCGTACGCACGCATAC
GCACACGCACGCACGCATATACACATATACACGCACACACACAACACACACACACACACTATATATATATATATA
TATATATATATATATATATATATATATATATATATATATATATATATATATATACATATATATATACATATATATATATATATATATA
TATATATATATATATGTATGTATGTATGTATATATATACATAAATACATAACCTTAAAACATACACATGTACAACCAG
AAAAGGCAGACAAAAAGACGCACACGCGACGTATTCTTTTTTTATTGTCATTATTCATCCATTCTCTTATCACA
CAAATGCTAGTCATCGCCGCCAGACTACTCACTCAGAATCTAAAAACAAGAAATTGGGAAACGTCACCAAAT
TAACGTCGAAAAGTGAAAGAAAGACATATAGATATAGTTTATATACACATATATATATATATATATATATATATATAT
ATATATATGTATAAATATATATATATATATATATATATATATATATATATATATATATATATATATGAATATGTATATATAATATATATA
CATCCATACATACATATAGATAGATAGATAGATAGATAGATAGATAGATAGATAGATAGATAGATAGATAGATAG
ATAGATAGATAGATAGATAGATAGATAGATATCTGTGTGTGTGTGTGTGTGTGTGTGTGTGTGTGTGTGTGTGT
GTGTGTGTGTGTGTGTGTGTGTGTGTGCGTATGCATATAGATCCTCCATCTCACTACTTACAGTTATCAGCCATC

GTGTCTTTCTCAACGTCCCCGCGTGTCCTAATAATCCGATCTTGATATCATCACTATGCGATTGTCCCCCGAAGT
ATGACGTCACGCGGCATCCCCCAAAGATGAGCGTGAGTCACCGGCAGTCACGGAGGCCAGCTCCTCGTCAT
CCTCCCGCCGGATTCGCGGGAAAAAGTGAAAGTCTTCCAAATATGGAGGCGTTTTTCCCCTATATTTAGCATC
ACTTGCCATGACGTTTTTATTTTTGTTATCATTTTTACTATTATTCTTAATAATACTTCTGTTTATCATTATTCTATT
TATCATTGTTGTTATGATCGTTATCATCATCTTCATCATTATAATCATTAATATGGCTTATTATTATTAACGCTACCG
TCATCCTCATCCTCATCATCCCACCTCCACGAACATCACACTCCCCAACAAAGCATGAGCCCCCCTCTCCCTGG
GACGTCACCAACACCCCCCGCAACCTTCACCCTCGCGAAGCAGCATGTCTCCCGCACGCCGCGAGGTCCAC
TTGGCCTGACCCCGTCGCCCCCTCCTGCAGGTGTGCTCCGCCGCCCTGGTGTCGCTGCTGGTGCTGGCCCTG
TCGTCCCGCAGCGCCTTCGCCCGCTCCGTCGACGGCGTGGGGCGCCTTGAGAAGCTGCTGTCGTCCTCGTC
GTCTTCGTCAGGCTCTTCTTCCCCCCTGGATGCTCTCGGCGGCGACCACAGCGTGAACAAGCGCGACACCTT
CGACCACTCCTGCAAGGGCATCTACGACCGGGAGCTCTTCAGAAAGCTGGACCGCGTCTGCGAGGACTGCT
ACAACCTGTACCGCAAGCCCTACGTGGCCACCGAGTGCAAGTAAGAGCCGCGGATTCTGGCACTTTGTTCT
CCTGTCTCTCTCTCAGTTTGTCTCACCATTTAAGTTGATTCTTATATAAGACGGGTTATTTTGTTTGACTTATTTT
CTGTTATGTTCTTTTTTTTACGTTTTAGTTGCTTTTATTTCTTCTTTTATTTATAATGATAATTTGTACATTACATTG
TTCTTATTTTTTTCTCTTTTTCCCATTCCTTCTCCCTTTACGAAAAAACAGGTCCAATTGCTTCGTGAATAAGAG
GTTCAATGTCTGTGTGGCTGATCTCAGACATGATGTCAGCCGCTTTCTGAAAATGGCTAAATTTCTGCGCTAT
CCCTAATGGTTGAAGGCTATGGTAATTTTATGTTTTCTTTTTCGTTTGACTATTTTTAGTTAGTGTTTTTAGTTT
TTTTTTTTTTTTTTTTTTTTTACCTATTATCATTTTTTGCATTTTAAAAATTATTTTCCAATTTTATTTGCAAAGAC
TGTCATCGCTTCTCTTCACCATATTTCCCATGGCTTTCCGCAACGATTATCAGACATTACCTGGTTTCTTTCCTC
TGAATTAATTTGTCATCATTTAATTTAGTTAACCCCTAAAGAATTTTCTTTAATGCAACCAACTCTAATAGAAAA
CGGGTTCTTGCTTTTCAGGAGTAACTGCTACGCCAACTTCGTATTCAAGCAGTGCCTCGACGATCTCCTTATG
GTCGACGCCATTGACGAGTACGTGAACACCGTCCAGCTGGTAGGGAAGTAAAGGCAGAAGTCTCTCAGGA
CGCTAATGTGGAGGAAACAAGAAAAAAAAAACAGGAAAAATATGGAAATTTTCACGACAACTAGAATCATTT
TTGAAAGCCCCTTGTCTGGAGACTTGGGCAAAATGGGGCCAAAGAAAATAGAGATGCAGTAAAGAATTATT
TTAAGTTGTCTTCTTCAGGAAAACATGACGCTCTATGGCAGCTCCTTTATCATTCCATACTAATTAGTAAAATAT
GATTTCTACCTAGTTATGAAATACATAGTAACTATATTATGCTAAGTATTTATTGAATATCATATATTTATTTTCATA
ACCTTTTGAAGTGGCATTTGAACGATTCTCACCACTGTCCTTCTCCCATTCATTTCTTCCTTCTCCCTCTCCTCA
ACTATTTATGCAATTCTGGTTATACGCTTTGAAAAGTTATTTTTGTATGGTCGATAATCATTAATCATAATACAGT
GTACAATTCGGCATTTATGTGTGAAAGAAAGGCTAGGAAACCACACAGCACATGAAGGCACGTAAGATTCA
GTATGGTTTCTTATCTGCGGCCTGTTGGGATCTGAAAATTATATTTTCTTGAATCTGTAGACCCCCTTCGCACA
AAATGAGGTAAAGTTACAAAGTTCTGAGCTGGAAGAAAAATCCAGTGCTACTTTGTCTTTCCATTCCCCTACC
CTTTCCCCCACACAGAGGGGGGGGGGACACAGCGATTTGCTTGCTAACTTCCACAAAATCAGATCTTTCCC
CTTTGAAAATTCTTGTTGTTTCAGTCGGTTGAGTCTTTTAGAAATAATATCTTCCCCAACTCGAAGCAAGGTAT
TCTTCAGCTTCTTAAGTAAAAAGAGGAAAGGCACGATCACCTGCTTTTCCAAATCAAGAGAAAGAGAATGC
GAAAGACAGAGAAATTCTTGATCTCTGTACTTACCACTTGTTGCTGGTGGTGCGTGTGGTGCCCGAACCCTA
GTTATGTAACCAAAGATTAGAATTTAGTGGTATCCTTAACTTTTGCGTTTCTGTTGGATCCTTGAGATTTTGAA
TAAATTATTATAATCA

Sequence of LvCHH Ia-1

>LvCHH Ia-1 protein

MVSFLSLRMVCSAALVSLLVLALSSRSAFARSVDGVGRLEKLLSSSSSSSGSSSPLDALGGDHSVNKRDTFDHSCK
GIYDRELFRKLDRVCEDCYNLYRKPYVATECKSNCYANFVFKQCLDDLLMVDAIDEYVNTVQLVGK

>LvCHH Ia-1 mRNA

ATGGT**CAGCTTCCTCTCACTTCGCA**TGGTGTGCTCCGCCGCCCTGGTGTCGCTGCTGGTGCTGGCCCTGTCG
TCCCGCAGCGCCTTCGCCCGCTCCGTCGACGGCGTGGGGCGCCTTGAGAAGCTGCTGTCGTCCTCGTCGTC
TTCGTCAGGCTCTTCTTCCCCCCTGGATGCTCTCGGCGGCGACCACAGCGTGAACAAGCGCGACACCTTCG
ACCACTCCTGCAAGGGCATCTACGACCGGGAGCTCTTCAGAAAGCTGGACCGCGTCTGCGAGGACTGCTAC
AACCTGTACCGCAAGCCCTACGTGGCCA**CCGAGTGCAAGAGTAACTGC**TACGCCAACTTCGTATTCAAGCA
GTGCCTCGACGATCTCCTTATGGTCGATGCCATTGACGAGTACGTGAACACCGTCCAGCTGGTAGGGAAGTA
A

Sequence of LvCHH Ia-2

>LvCHH Ia-2 protein

MVSFLSLRMVCSAALVSLLVLALSSRSAFARSVDGVGRLEKLLSSSSSSSGSSSPLDALGGDHSVNKRDTFDHSCK
GIYDRELFRKLDRVCEDCYNLYRKPYVATECKSNCFVNKRFNVCVADLRHDVSRFLKMAKFLRYP

>LvCHH Ia-2 mRNA

ATGGTCAGCTTCCTCTCACTTCGCATGGTGTGCTCCGCCGCCCTGGTGTCGCTGCTGGTGCTGGCCCTGTCG
TCCCGCAGCGCCTTCGCCCGCTCCGTCGACGGCGTGGGGCGCCTTGAGAAGCTGCTGTCGTCCTCGTCGTC
TTCGTCAGGCTCTTCTTCCCCCCTGGATGCTCTCGGCGGCGACCACAGCGTGAACAAGCGC**GACACCTTCG**
ACCACTCCTGCAAGGGCATCTACGACCGGGAGCTCTTCAGAAAGCTGGACCGCGTCTGCGAGGACTGCTAC
AACCTGTACCGCAAGCCCTACGTGGCCACCGAGTGCAAGTCCAATTGCTTCGTGAATAAGAGGTTCAA**TGTC**
TGTGTGGCTGATCTCAGACATGATGTCAGCCGCTTTCTGAAAATGGCTAAATTTCTGCGCTATCCCTAAGAGT
AACTGCTACGCCAACTTCGTATTCAAGCAGTGCCTCGACGATCTCCTTATGGTCGATGCCATTGACGAGTACG
TGAACACCGTCCAGCTGGTAGGGAAGTAA
