# Supplementary material for: Identification and Functional Analysis of a Key Gene in the CHH Gene Family for Glucose Metabolism in the Pacific White Shrimp Litopenaeus vannamei
Source: Int J Mol Sci. 2025 May 12;26(10):4612. doi: 10.3390/ijms26104612 (PMC12111282; doi:10.3390/ijms26104612)
Supplement: Supplementary file 1 [file ijms-26-04612-s001.zip › Supplementary Figure S1-S5.pdf]

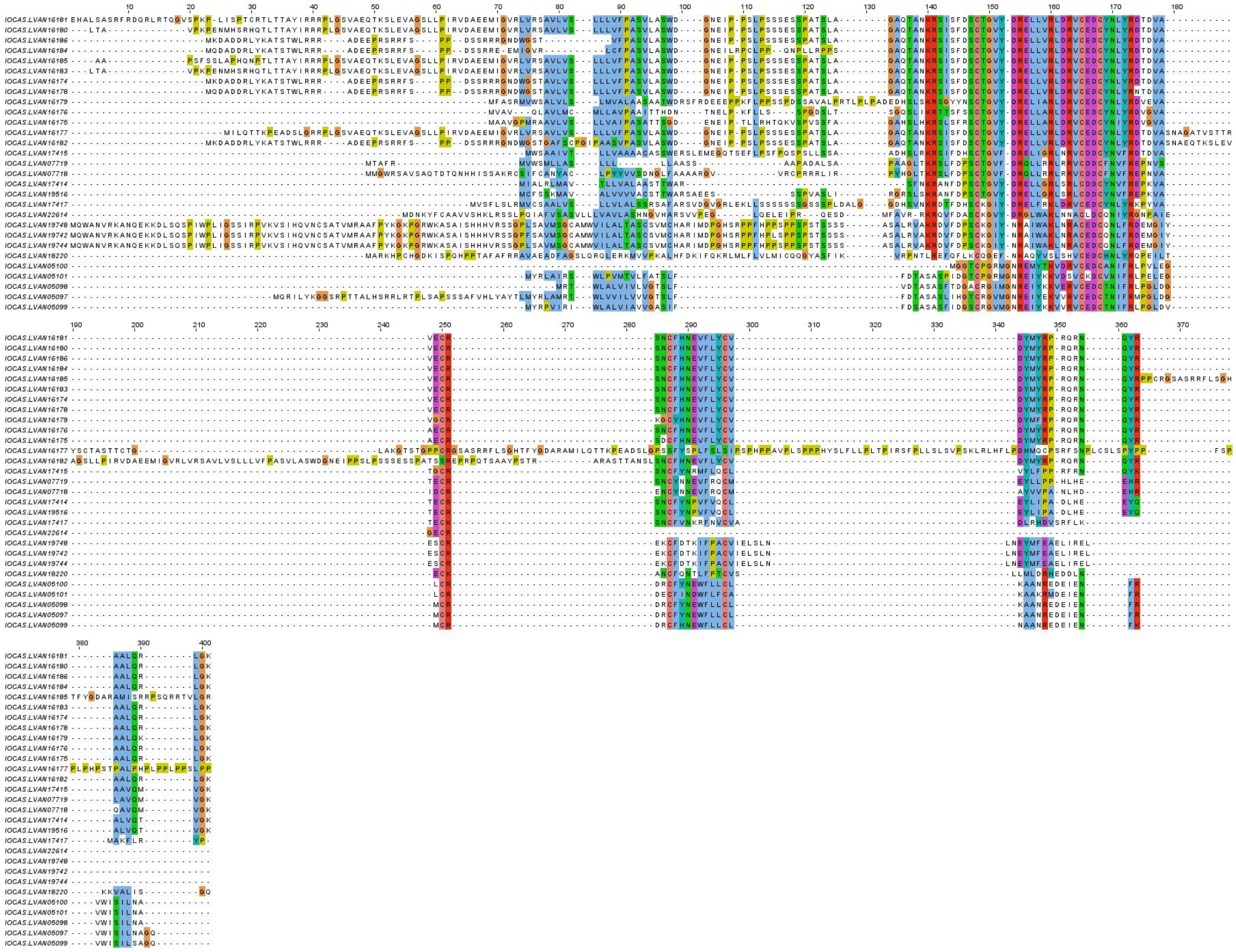

Supplementary Figure S1. Sequence alignment of the ORF regions of 29 CHH genes.

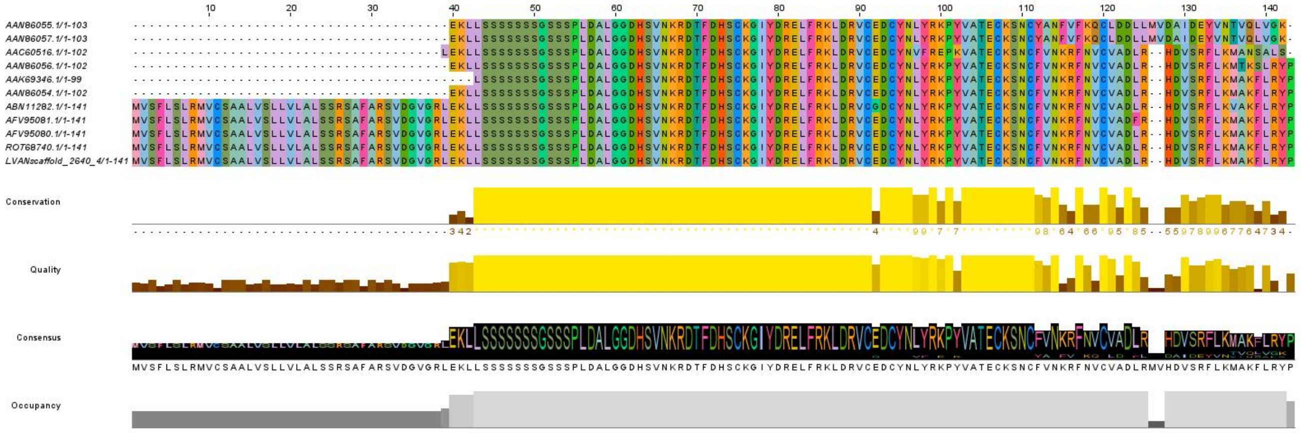

Supplementary Figure S2. Amino acid sequence alignment of CHH Ia protein.

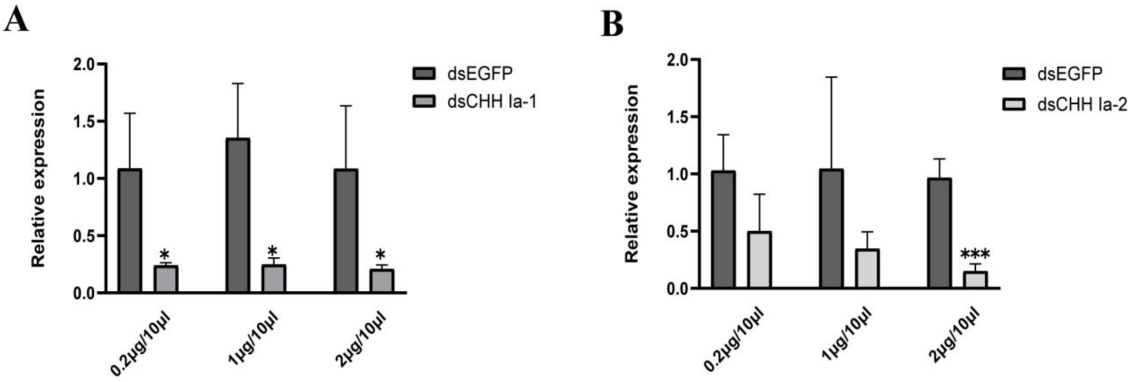

Supplementary Figure S3. RNA interference preliminary experiment interference efficiency detection. (A)dsCHH Ia-1interference efficiency detection. (B)dsCHH Ia-2interference efficiency detection.

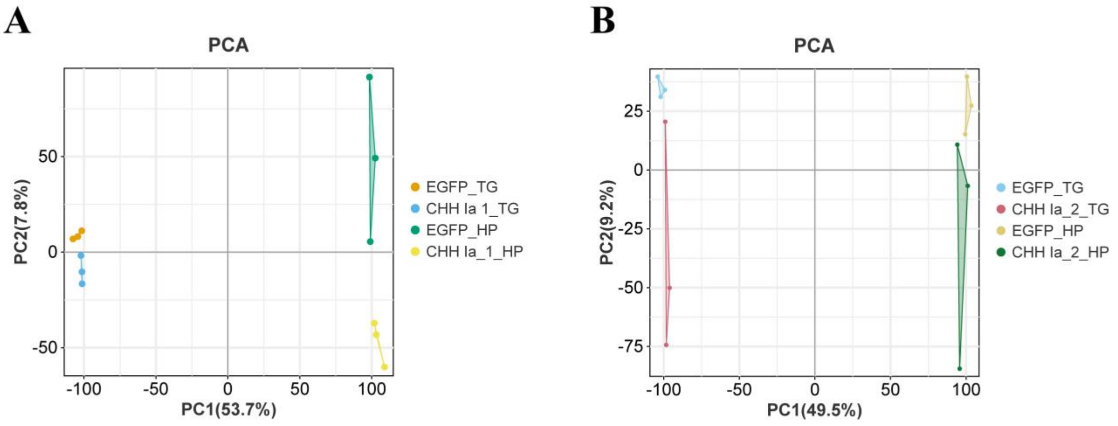

Supplementary Figure S4. Transcriptome PCA analysis. (A)ds CHH Ia-1interference group. (B)ds CHH Ia-2 interference group.

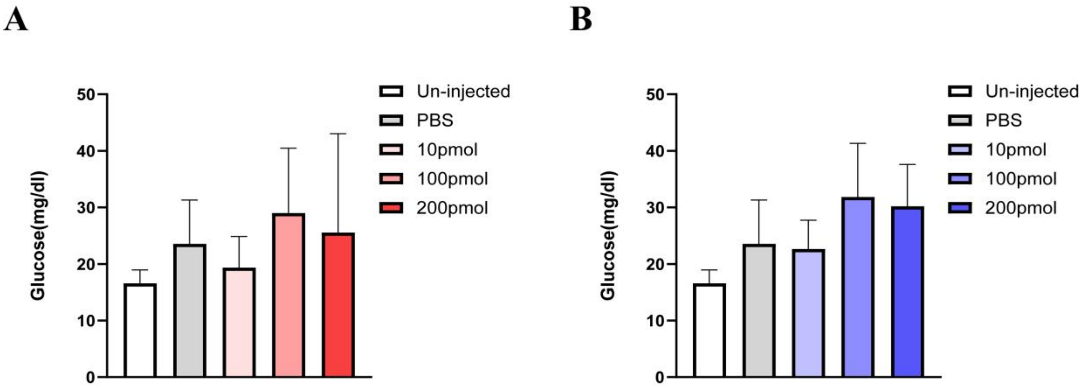

Supplementary Figure S5. Changes in blood glucose induced by injection of rCHH at three different concentrations. (A)Changes in blood glucose 1h after rCHH Ia-1 injection . (B)Changes in blood glucose 1h after rCHH Ia-2 injection.
